# Corrective osteotomy of distal radius malunion after IIIB open fracture: Palmar approach - Case report

**DOI:** 10.1016/j.ijscr.2019.04.020

**Published:** 2019-04-28

**Authors:** Cen Bytyqi, Hasime Qorraj, Arber Tolaj, Rrahman Hajdari

**Affiliations:** a Medical Faculty, University of Prishtina “Hasan Prishtina”, Kosovo; b University Clinical Center of Kosovo, Orthopedic Department, Kosovo; c Regional Clinical Center of Gjilan, Kosovo

**Keywords:** Open fracture type IIIB, Malunion, Corrective osteotomy, Case report

## Abstract

**Background:**

Despite the fact that distal radius fractures are the most common, open fractures are relatively uncommon. The aim of this study is to present the case with severe malunion of extra-articular left distal radius after open fracture grade IIIB (Gustillo). The treatment was done with open correctuve osteotomy and ostosynthesis with volar low profile plate.

**Case presentation:**

A 32-years old man was admitted to our departement because of severely malunited left distal radius extra-articular metaphyseal fracture after treatment of an open fracture. He was primary treated in a Regional Hospital with irrigation, debridement, antimicrobial cover, and immediate skeletal management through fixation with Kirschner’s wires. No external fixator was used. He developed volar metaphyseal angulation after loss of primary reduction of K-wires fixation; therefore it required corrective surgical treatment. Because of malunion, open corrective osteotomy and bone grafting of distal radius with volar plating were done. Follow-up 20 months after surgery show deformity correction of radial volar inclination, height, and restitution of distal radioulnar joint.

**Conclusion:**

This case report offers an informative overview on this uncommon case of distal radius open fracture malunion treated successfully with volar approach corrective osteotomy. Corrective surgery is the primary therapy of choice in the this case. This method have shown to be safe and effective in treatment of distal radius malunion with volar angulation.

## Highlights

• This is a rare case of Type IIIB open distal radial fracture malunion.
• The fracture was primary treated with fixation with Kirschner’s wires.
• The patient has developed volar metaphyseal angulation after loss of primary reduction of Kirschner’s wires fixation.
• Volar approach for corrective osteotomy and plate insertion is an effective treatment of fracture malunion.

## 1 Background

Distal radial fracture is the commonest type of fractures in adults, constitutes 17% of all fractures, but open distal radial fractures are relatively rare [1,2]. Open fractures of distal radius are high-energy injuries that are presented with spectrum of soft tissue and bone injury. This is usually associated with massive contamination.

Unstable and extra articular open fractures of the distal radius remain a challenging problem in management. A good primary management is very important especially due to the possibility of multiple type of complications. Several methods of treatment have been described in the management of these conditions, such as reduction and pinning, external fixation etc. [3,4]. A key method for surgical fixation is percutaneous pinning, involving the insertion of wires through the skin to stabilise the fracture. Reduction and retention, however, may deteriorate during and after application of Kirschner’s wires osteosynthesis. Caused by collapse, loss of normal palmar tilt, radial shortening, articular incongruity and permanent deformity with pain and loss of function may result. Volar and dorsal angulation of radiocarpal joint surface worsen functional outcome considerably when it exceeds 20 ° [[3], [4], [5], [6]]. In traumatic pathology, two types of malunions of the distal radius should be distinguished: extra-articular and articular. Extra-articular malunion after treatment of open distal radius fracture is relatively rare complication. Malunion of the distal radius is particularly problematic at the distal radioulnar joint. As radial height is lost, radio-ulnar length mismatch may result in distal radioulnar joint incongruity, causing instability, decreased motion, and early arthrosis [[7], [8], [9]]. However, there is a controversy about the method of treatment of extra-articular distal radius malunion after open fracture.

This study is reported in line with the SCARE criteria [10] and was designed to evaluate the outcome results in treatment of the extra articular open fracture Gustilo type IIIB of the distal radius with extensive soft tissue injury, periosteal stripping and bone exposure.

## 2 Case presentation

**Fig. 1:**
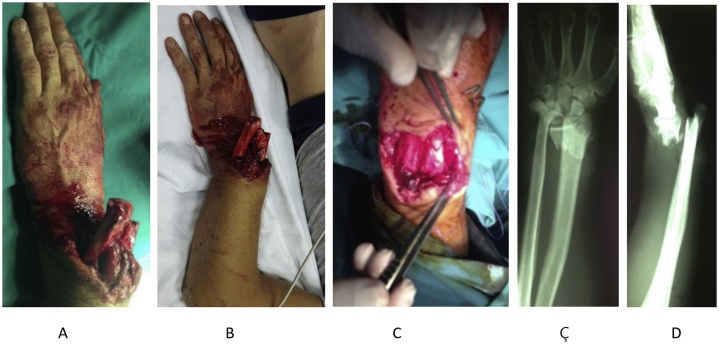
Preoperative images of a 32-year-old man left wrist with severe deformity. A, B, C, D: Pictures obtained on the date of injury. The patient sustained an open extra-articular distal radius fracture with large dorsal soft tissue defect type IIIB.

A 32-year - old man sustained an open distal radius fracture (AO 3 A, Gustillo IIIB). Primary treatment was done at the Emergency Department of a Regional Hospital, where the patient received intravenous antibiotics, sterile dressing and cast longuette immobilization. Within 4 h, the patient underwent debridement, reduction and operative fixation with three Kirschner’s wires and plaster immobilization. The extrenal fixator was not used. Following the fixation of the fragmented fractures, myorrhaphy-tenorrhaphy on the injured tendons was done (Fig. 1).

**Fig. 2:**
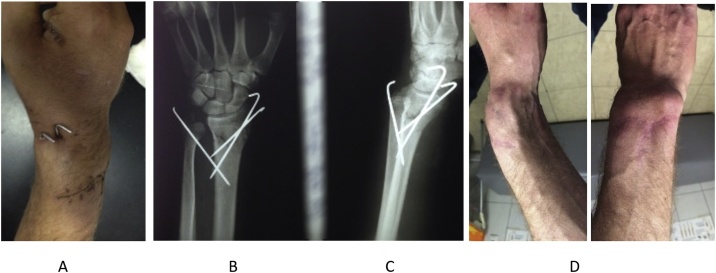
A: Three weeks postoperatively, reduction is still saved. B: Five weeks postoperatively X- Rays, dislocation and volar angulation deformity. Eight months after the injury, clinical pictures in sagital and frontal plane showed a volar angulation deformation of left distal radius.

The patient was presented in our department, in University Clinical Center after 8 months of injury. After loosening of the primary reduction and fixation, redislocation occurred and malunion was developed. The patient lost normal volar tilt, radial inclination, and radial length relative to the ulna and complained of pain, limited motion, decreased grip strength, and cosmetic deformity (Fig. 2)

**Fig. 3:**
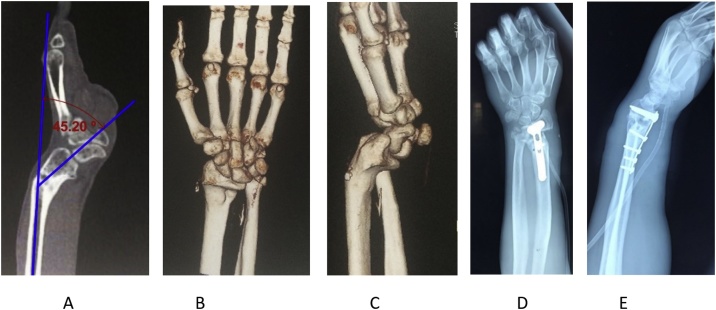
A: The patient’s preoperative CT scans showing malalignment of the distal radius in both sagittal and coronal planes. A: The angle of volar inclination is 45 ° in sagital plane. B: CT scan with reconstruction in the coronal plane and C: in sagital plane. D, E: The first postoperative plain radiographs in a-p and lateral view showing deformity correction.

The distal radioulnar joint was also dislocated and therefore prono-supination was limited. Because of the conflict of the ulnar head with the carpus, patient developed an ulnar painful wrist. CT images with 3D reconstructions were used for pre-operative planning (Fig. 3A–C) and corrective osteotomy and fixation with volar plate was planned.

Surgical technique

The aim of corrective osteotomy was to achieve anatomic relation in the wrist joint obtaining stable fixation with minimal disruption of supporting ligaments and with maintance of the vascularity of the fragments. The contralateral unaffected radius was used as a reference in planning of a corrective osteotomy procedure of a malunited distal radius. The planning was arranged with a volar approach and using a volar plate as a fixation device after the osteotomy.

The patient was placed in the supine position on operating table with the forearm on a hand table in supinated position and the palm faced upward. Exsanguinating bandage was used. A 6-cm longitudinal incision was made over the flexor carpi radialis tendon proximal to the wrist flexion crease. The exposure was done with a great care, atraumatic and respect soft tissues. Fluoroscopy images were taken to assure appropriate position of the plate and for planning of the osteotomy. The position of the plate and osteotomy were marked but the distal holes were not predrilled. The plate was then removed and an osteotomy was performed closely to the original fracture site and just proximally to the sigmoid notch. It was attempted to place the transverse metaphyseal osteotomy at the site of deformity and roughly perpendicular to the shaft of the radius. Trapezoidal corticocancellous autologous appropriate size and shape from the iliac crest was used and placed in the bone gap for its osteoinductive potential. The plate was then fixed to the distal fragment to allow appropriate correction of volar tilt and radial inclination. Image intensifier fluoroscopy was then used to assess plate position, and adjustments to radial and volar inclination and height. The patient postoperatively was placed into a well-padded, above-elbow splint.

## 3 Postoperative evaluation

**Fig. 4:**
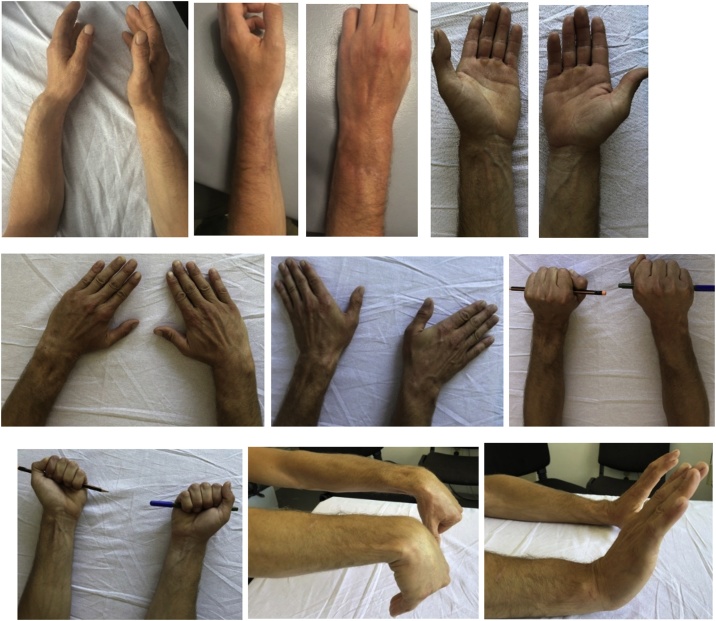
Postoperative follow-up.

**Fig. 5:**
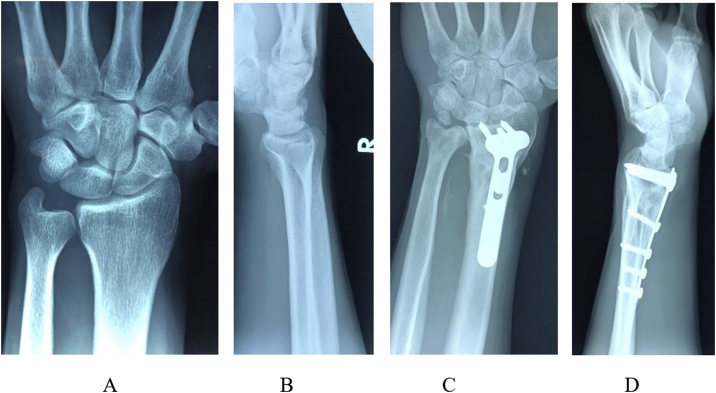
Final follow-up of a patient treated with palmar aproach, 20 months postoperatively. A, a-p view of the uninjured right wrist shows an ulna slightly minus variance. B. Lateral view of the normal uninjured right wrist. C, D. Postoperative X - rays of the left wrist showing complete healing of the left distal radius after corrective osteotomy and restoring proper anatomic parameters comparing to the normal right side.

In order to have a better post operative evaluation, the objective measurements, examination of the wrist range of motion (ROM) and grip strength (with a dynamometer-Jamar) were done (Fig.4). The quantification of these measurements show approximation of results with normal rates (Table 1) (Fig. 5).

**Table 1:** Range of motion and Grip strength at last follow-up visit for injured and normal wrist.

Wrist motion And Grip Strength	Dorsal flexion	Palmar Flexion	Radial deviation	Ulnar deviation	Supination	Pronation	Grip Strength-Jamar (kilograms)
Injured (L)	74	75	21	31	82	85	49
Normal (R)	80	82	24	35	90	90	55

Also, 20 months postoperative X - rays of the left wrist show complete healing of the left distal radius after corrective osteotomy and restoring proper anatomic parameters comparing to the normal right side.

## 4 Discussion

Anatomical reduction after extra-articular open fracture of distal radius is the principal aim of treatment but cannot achieve long term retention of fragments with simple fixation with Kirschner wiring.

Kurylo and colleagues [11] reported that immediate open reduction and internal fixation with plates may be safe in grades I and II injuries. Rozental and colleagues [3] documented increasing injury severity by Gustilo classification was associated with a greater number of poor results and postoperative complications.

Nyquist and Stern reported decreased range of motion and wrist pain with activity for all of their patients with open fractures at final follow-up [12]. Rozental et al. found decreased range of motion (avg. flexion, extension, supination, and pronation: 37°, 40°, 47°, and 57°, respectively), decreased grip strength (50.6 lbs), and high levels of perceived disability (avg. DASH score 33) in their study patients twenty-four months post-injury [3].

There doesn’t exist a conclusion about the difference between external fixation and plating. Many surgeons likely feel comfortable with plating at the time of initial debridement, based on both experience and the literature supporting immediate internal fixation after initial debridement in open forearm and hand fractures [[3], [4], [5],^11^].

An important issue in the treatment of open radius distal metaphyseal fractures is redislocation and malunion.

Distal radius malunions frequently have an unappreciated rotational component that may affect distal radioulnar joint (DRUJ) motion and stability. In a study that examined computed tomography (CT) scans of both affected and normal wrists, Prommesberger et al. reported a rotational component to the malunion in 23 of 37 patients [13]. When volar angulation of the distal articular surface of the radius becomes greater than 25°- 30° in the sagittal plane, like in our case, corrective osteotomy is recommended. Corrective osteotomy of the distal radius with signiﬁcant malalignment after a fracture is an option to restore the functional anatomy of the wrist, but can be a difﬁcult procedure anyway [14,15]. The ability of an osteotomy to restore alignment depends on the location of CORA, the axis about which correction is performed (the correction axis), and the location of the osteotomy. Mulders et al reported that corrective osteotomy is an effective method of treating symptomatic distal radius malunions with good long-term functional results, and improvement in radiographic parameters and pain scores [16].

## 5 Conclusion

In conclusion, we can say that this surgical technique to address distal radius malunions allows excellent correction of deformity, with low morbidity and no need of hardware removal.

Also, mechanical advantages offered by locking plates for treatment of acute fractures of the distal are attractive for corrective osteotomies as well.

Outcome scores as well as pre- and postoperative range of motion and grip strength tests document significant improvements in function.

## Conflicts of interest

No conflict of interest.

## Sources of funding

No source of funding.

## Ethical approval

The study is exempt from ethical approval in our institution.

## Consent

The patient was informed and agreed that his clinical data will be used for research purposes.

## Author contribution

Cen Bytyqi: Data Curation, Conceptualization and Supervision.

Hasime Qorraj: has drafted the manuscript and contributed to the literature review.

Arber Tolaj : Data Curation.

Rrahman Hajdari: Data Curation.

## Registration of research studies

researchregistry465.

## Guarantor

Cen Bytyqi.

## Provenance and peer review

Not commissioned, externally peer-reviewed.
